# Enhanced biocrude production from hydrothermal conversion of municipal sewage sludge *via* co-liquefaction with various model feedstocks

**DOI:** 10.1039/d2ra02325c

**Published:** 2022-07-13

**Authors:** Wenjia Wang, Hongbiao Du, Yuanyuan Huang, Shaobo Wang, Chang Liu, Jie Li, Jinglai Zhang, Shuai Lu, Huansheng Wang, Han Meng

**Affiliations:** Department of Chemical Engineering, University of Utah Salt Lake City Utah 84112 USA; School of Environment and Natural Resources, Renmin University of China Beijing 100872 China ls2020@ruc.edu.cn; High-Tech Research Institute, Beijing University of Chemical Technology Beijing 100029 China; Shenergy Environmental Technologies Co., Ltd Buiding 7. Vanka Canter, 988 Shenchang Rd Shanghai 201100 China hanmengcn@outlook.com

## Abstract

Hydrothermal co-liquefaction has the potential to improve biocrude yield. To investigate the influence of various types of biomass on co-liquefaction with municipal sewage sludge (MSS), experiments on MSS with three kinds of model feedstocks (soy oil, soy protein, and starch) were carried out. Reaction temperatures of 300, 320, and 340 °C proved to be the appropriate reaction temperatures for the highest biocrude yield for soy oil, soy protein, and starch, respectively. A synergistic effect on the biocrude yield of co-liquefaction was proved, and starch showed the highest synergistic effect with a 57.25% increase in biocrude yield, while soy oil only presented a slight synergistic effect. Thermal gravimetric analysis (TGA) results suggested that co-liquefaction with soy oil increased the light oil fractions in biocrude by 20.81%, but protein and starch led to more heavy oil fractions. Gas chromatography-mass spectrometry (GC-MS) indicated that co-liquefaction with protein or starch produced more cyclic compounds in the biocrude, while almost no new components appeared from co-liquefaction with soy oil.

## Abstract 2

Hydrothermal co-liquefaction has the potential to improve biocrude yield.

## Graphical abstract

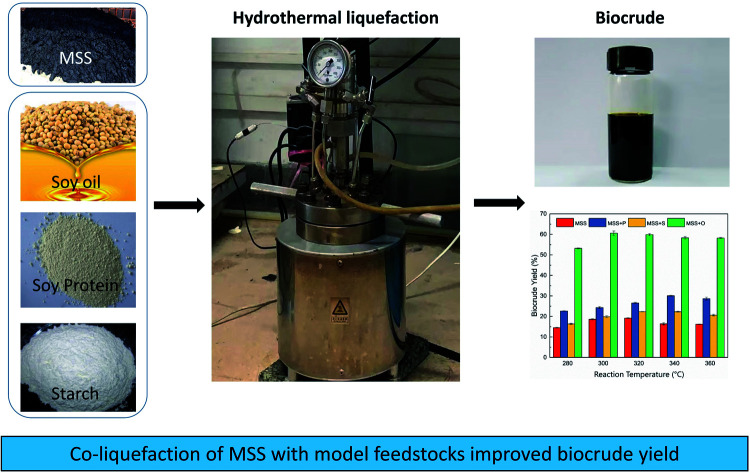

## 1 Introduction

With the fast-developing urbanization in China, the number of wastewater treatment plants (WWTPs) and the wastewater treatment capacity grow rapidly. Currently, more than 3500 WWTPs are busily treating the urban wastewater and discharging more than 6 million tons of dry municipal sewage sludge (MSS) every year.^1^ MSS, as the byproduct of the biological wastewater treatment process, contains various bacteria and parasites, as well as toxic and harmful heavy metals. As one of the most important municipal solid wastes, how to handle the disposal of MSS has become a hot environmental topic recently.^2^ However, conventional MSS treatment methods, such as landfilling, incineration, land application or ocean disposal, which could produce secondary pollution to earth, air, and water, have been proven to be less applicable to the greener requirements of modern society.^3^ Therefore, to build a more environmentally friendly wastewater treatment system, the current MSS treatment methods should be improved or replaced with advanced technologies.^4,5^ Meanwhile, MSS is rich in organic matter and could be a potential renewable raw material for sustainable biofuel production.^6^ Hence, the desired biofuel-producing conversion process should be able to handle the disposal and recycling of MSS biomass waste simultaneously.

Various valuable biofuels can be produced from MSS, like biogas, biodiesel, solid briquettes, pyrolysis bio-oil, and biocrude, *via* different biological, chemical, and thermochemical methods.^7–10^ Thermochemical conversion (liquefaction, gasification, pyrolysis, and carbonization) is believed to be the quickest pathway for biofuel production. Among these thermochemical methods, hydrothermal liquefaction (HTL), through which the bio-polymeric compounds in biomass are dissolved, hydrolyzed, and transformed into biocrude and other byproducts (aqueous products, gaseous products, and solid residues) in subcritical/supercritical water and an oxygen-free atmosphere, shows an excellent prospect.^11,12^ The HTL process is advantageous in saving on the feedstock drying cost, requiring a lower heating energy input, and reducing the requirement for special facilities, compared with other thermochemical conversion processes (pyrolysis, gasification, and carbonization).^13,14^ Moreover, the HTL operating conditions provide a sub/supercritical water environment to kill pathogens and passivate the heavy metals in MSS.^6^ Hence, the HTL conversion of MSS could be a promising method for the disposal and recycling of municipal sludge waste.

In the past few years, the HTL of MSS and the influence of its operating parameters (reaction temperature, holding time, solid ratio, and heating rates, *etc.*) were discussed in detail.^15^ However, the biocrude yield from the HTL of MSS still has room to improve, and the properties of the biocrude have not matched those of petroleum.^16^ To increase the biocrude yield and improve the quality of the biocrude, different improvement methods were introduced into the HTL of MSS. Adding homogeneous catalysts, involving an organic co-solvent, providing hydrogen donors, or reducing the atmosphere, could produce more and better biocrude, however, this requires a higher cost to be paid.^1,5,17–20^ Thus, attention was paid to the co-liquefaction of MSS and other organic biomass wastes from our daily life.^21^

As is widely known, the organic composition of the liquefaction feedstock is important for the HTL process.^22^ Co-liquefaction between various kinds of biomass has provided a promising way to improve the biocrude yield and properties.^23^ For example, co-liquefaction of microalgae and swine manure could increase the biocrude yield.^24^ Co-liquefaction of microalgae and macroalgae showed a deoxygenation effect on the obtained biocrude.^25^ Adding rice husks decreased the acidity and nitrogen content of microalgae-derived biocrude.^26^ And spent coffee grounds showed a synergistic effect for liquefaction with lignocellulosic biomass.^27^ However, limited information about the co-liquefaction of MSS with other kinds of biomass has been reported. A few previous works mainly focused on co-liquefaction of MSS with forestry and agricultural residues, which mainly consisted of lignin-derived compounds.^4,28–30^ Nevertheless, the composition of the liquefaction feedstock can affect the biocrude yield and its properties, while the biomass is a collective concept consisting of various types of organic matter with diverse biochemical compositions. In this case, the effect of other major kinds of organic biochemical compounds (proteins, lipids, and saccharides) on the co-liquefaction with MSS should be taken into consideration. Such research work could be beneficial for the selection of an appropriate co-liquefaction partner for improvement in the energy and resource recycling of MSS *via* the HTL method.

In this study, co-liquefaction of MSS with different model biochemical substances was explored. The main objective of this research was to investigate the influence of different compositions (proteins, lipids, and saccharides) on the co-liquefaction with MSS, and the possible synergistic effects between these feedstocks. Characterization of the composition and properties of the products was demonstrated. This study could help in the selection of an appropriate co-liquefaction partner during the HTL of MSS and provide a deeper understanding of the co-liquefaction of MSS.

## 2 Materials and methods

### 2.1 Materials

The MSS was collected from the secondary sedimentation tanks in Qinghe WWTP (Beijing, China). The obtained MSS was an activated sludge with a moisture content of around 80%. The content of ash, lipids, saccharides, and proteins was 27.00, 6.43, 33.25, and 33.32%, respectively. The soy protein, soy oil, and starch were purchased from Aladdin Reagent Co., Ltd (Shanghai, China). The detailed composition of the feedstock is presented in Table 1. Deionized water and ethyl acetate (analytical pure, Beijing Chemical Works) were used in the HTL process. All the other reagents used in the characterization were analytical grade pure.

**Table 1:** The proximate and ultimate analysis of the MSS and model feedstocks

Compounds	MSS	Soy protein	Soy oil	Starch
Ash content (%)	5.02	—	—	—
Moisture content (%)	81.42	—	—	—

**Organic element content (%, daf** a **)**				
C	46.9	44.5	65.2	38.5
H	7.0	6.5	9.8	6.0
O^b^	37.9	35.2	24.9	55.5
N	7.5	13.8	0.1	0.0
S	0.7	0.0	0.0	0.0

**HHV (MJ kg** ^**−1**^ **)**				
—	21.54	20.63	31.93	15.10

a Daf: dry ash free.

b Calculated by differences.

### 2.2 Co-liquefaction operation and product separation process

The HTL experiments were carried out in a high-pressure batch reactor made of 316 stainless steel with a volume capacity of 2.0 L (GS-2.0, Weihai Chemical Machinery Co., Ltd., China). In a typical HTL run, 40 g of dry MSS, 40 g of model feedstock (soy protein, soy oil, or starch), and 320 mL of deionized water were fed into the reactor. The reactor was sealed and pumped with pure nitrogen gas to expel the air in the reactor and provide an inert atmosphere. The sealed reactor was heated with an electric heating jacket. When the reactor reached the set temperature, which was defined as the reaction temperature, it was then held at that temperature for 30 min. After that, the reactor was cooled down with an inner-circulating water cooling system to room temperature (25 °C). In the liquefaction process, a series of experiments with the MSS alone were used for comparison and defined as the blank group.

**Fig. 1:**
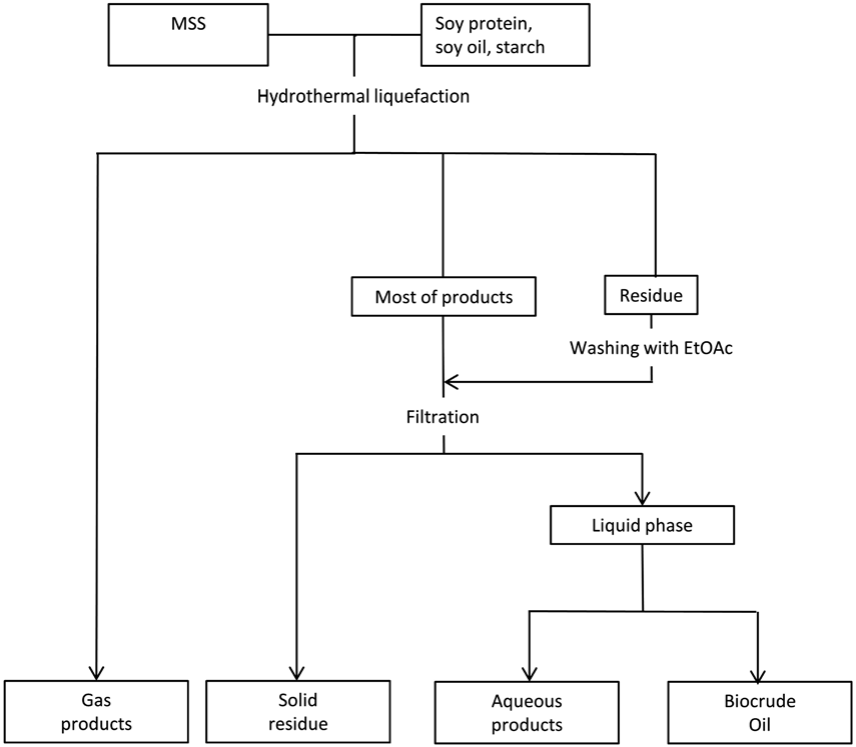
Separating procedure of HTL products.

The separating procedure for the HTL products is shown in Fig. 1, according to Yang’s^31^ research. The cooled reactor was depressurized through the vent valve, and the gaseous products were collected in a pre-weighted and pre-vacuumed gas bag. The weight of the gaseous products was measured with an analytical balance. The mixture of liquid products and solid residue was poured out, while the inner wall of the reactor was washed with ethyl acetate (EtOAc) to wash down all the products. The EtOAc washing liquid was mixed with the product mixture and then filtrated. The separated liquid was divided into two phases in a separating funnel: an EtOAc-soluble fraction and a water-soluble fraction. The EtOAc phase was treated in a rotary evaporator at 80 °C and 0.1 MPa. The obtained EtOAc-free black viscous liquid was designated as the desired biocrude product and weighed after cooling down.

The theoretical biocrude yield, biocrude yield and element enrichment were calculated by the following eqn (1)–(3):1
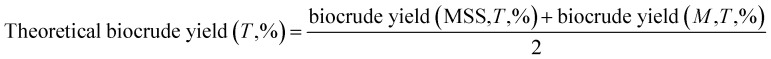
2
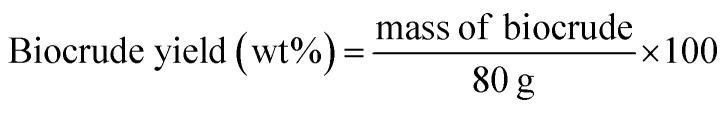
3
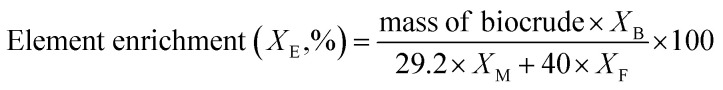
where *T* represented the reaction temperature of co-liquefaction, and *M* was the model biochemical compound (soy protein, soy oil, starch, or lignin). The theoretical biocrude yields were calculated from the mixture ratio of 1 : 1, and the experimental yields from the individual liquefaction of the MSS and the three kinds of model feedstocks, based on the assumption that no synergies existed during co-liquefaction. 29.2 g is the mass of organic matter in the 40 g of dry MSS, *X*_B_ is the element content percentage in the biocrude (*X* = C, H, O, N, and S). Similar abbreviations *X*_M_ and *X*_F_ are the element content in the organic matter of the MSS and in the model compound feedstock, respectively. F represents the soy protein, soy oil, and starch.

### 2.3 Biocrude characterization

The elemental compositions (C, H, N, and S) of the feedstock and the obtained biocrude samples were analyzed with an elemental analyzer (Vario EL). The content of elemental oxygen (O) was determined by difference. The analysis was repeated at least three times, and only the average value is presented. The higher heat values (HHV) of the feedstock and biocrude samples (eqn (4)), and energy recovery (ER) (eqn (5)), were calculated by the equations described in the previous literature:^32^

4 HHV (MJ kg^−1^) = 0.3404*C*_B_ + 1.2432*H*_B_ + 0.0628*N*_B_ + 0.1909*S*_B_ − 0.0984*O*_B_

5
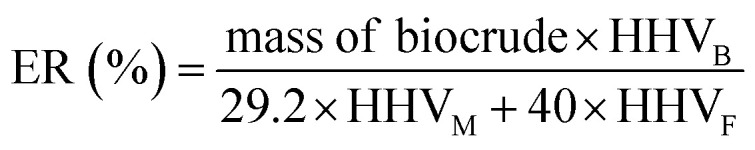
where *C*_B_, *H*_B_, *O*_B_, *N*_B_ and *S*_B_ are the mass percentages of carbon, hydrogen, oxygen, nitrogen, and sulfur elements in the biocrude samples, respectively. HHV_B_, HHV_M_, and HHV_F_ are the higher heating values of the biocrude, MSS, and model compound feedstock, respectively.

A TG analyzer (DTG-60, Shimadzu, Japan) was used for the thermal gravimetric analysis (TGA). Each sample (15 ± 0.5 mg) was heated from 50 to 500 °C at a heating rate of 10 °C min^−1^ in pure nitrogen gas with a flow rate of 5 mL min^−1^. Each experiment was replicated three times to ensure reproducibility and the average values presented.

The volatile components in the biocrude samples were analyzed by gas chromatography-mass spectrometry (GC-MS, QP 2010, Shimadzu, Japan). An Rtx-1701 capillary column (60 m × 0.32 mm × 0.25 μm) was used in the GC-MS analysis. A temperature program of heating to 40 °C, holding for 2 min, then ramping up to 250 °C was used. The identification of compounds was based on the NIST Database (NIST11).

## 3 Results and discussion

### 3.1 Effect of reaction temperature on biocrude yield

**Fig. 2:**
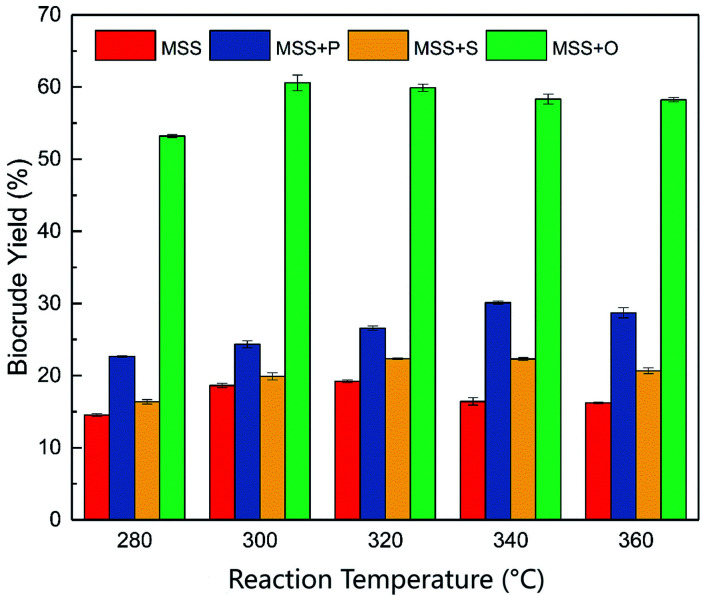
The biocrude yields of co-liquefaction under different temperatures. P: soy protein; S: starch; O: soy oil.

The reaction temperature is considered the most dominant operating parameter for the liquefaction of biomass. An appropriate reaction temperature can strongly improve the biocrude yield and properties.^33^ In this section, the effect of reaction temperature on HTL product distribution was investigated and presented in Fig. 2. The HTL experiments were performed with a holding time of 60 min and a solid percentage of 20%. The HTL reaction temperature ranged from 280 to 360 °C.

As shown in Fig. 2, the reaction temperature showed an apparent effect on the biocrude yield. The general trend is that the biocrude yield changed in two steps. In the first step, the biocrude yield gradually increased with higher reaction temperature. However, the co-liquefaction with various model feedstocks reached the highest biocrude yield at different reaction temperatures of 300 (for soy oil, 60.57%), 320 (for starch, 22.33%), and 340 °C (for soy protein, 30.12%). The large differences in biocrude yield could come from the different biochemical compositions of the co-liquefaction reactants. It is believed that the conversion yields of the different compounds are ranked in the following order: lipids > proteins > saccharides.^34^ In the meantime, with a further increase of reaction temperature in the second step, the biocrude yield showed either a slight decrease or remained almost unchanged, depending on the species of the added co-liquefaction compounds. As is widely known, the macro-biomolecules (proteins, saccharides, and lipids) undergo a battery of different degradation reactions (hydrolysis, dehydration, condensation, decarboxylation, decarbonylation, deamination, *etc.*) and are cracked into a series of small molecules or intermediates.^35^ It should be noted that these different reactions of different compounds required different appropriate degradation temperatures.^36,37^ Previous research suggested that adding different liquefaction feedstock with different biochemical composition could change the biocrude significantly.^38^

According to Fig. 2, all three kinds of co-liquefaction compounds increased the biocrude yield in the experimental reaction temperature range, compared with the individual liquefaction of the MSS. Even so, a further comparison suggested that the performance of the various co-liquefaction feedstocks was different. The soy oil, which represented the lipids, gave the highest biocrude yield, while the starch, which represented the saccharides, provided the lowest improvement in the biocrude yield. The observed phenomena were consistent with the co-liquefaction of microalgae and other biochemical compounds.^21^ The different improving effect, however, could be associated with the difference in biochemical composition of the co-liquefaction compounds in the MSS. The lowest improvement from the saccharide (starch) could be due to the fact that saccharides are the most difficult components to be converted into biocrude during the HTL process, compared to other kinds of biomass compounds.^39^ It is well established that the reaction with saccharides requires a higher activation energy to form the biocrude.^40^ The highest biocrude yield from soy oil (lipids), on the other hand, could come from the easier conversion of lipid compounds than other kinds of biomolecules. Due to the complex structures of protein molecules, the decomposition of protein would be harder than that of lipid in the HTL system. That could be the explanation why the highest biocrude yield from co-liquefaction with the protein needed the highest reaction temperature of 340 °C. However, the HTL process that occurred at a higher temperature and a higher pressure proved to be a good method to convert proteins into biocrude.^41^ In that case, there is no doubt that adding extra protein could increase the biocrude yield effectively.

Overall, co-liquefaction of MSS with lipids, proteins, and saccharides, could promote the production of biocrude. However, the reason was still unclear. The increased biocrude yield could have come from free radicals or intermediates, produced by the degradation of extra biochemical components, which synergistically formed more biocrude. Meanwhile, one cannot deny that adding various organic feedstocks (proteins, lipids, or saccharides) into the MSS liquefaction system could be regarded as an increase in the organic content of the liquefaction reactants. Obviously, a higher concentration of liquefaction reactants might benefit the HTL process and lead to a higher biocrude yield. Therefore, whether the additional feedstock played a synergistic role in the co-liquefaction process or the extra biochemical compounds only increased the biocrude yield individually should be taken into consideration. At the same time, according to Fig. 2, reaction temperatures of 300, 320, and 340 °C were the reaction temperatures for the highest biocrude yield for the co-liquefaction of the MSS with soy oil, starch, and soy protein, respectively.

### 3.2 The synergistic effect of co-liquefaction with different biochemical compounds on the biocrude yield

During the co-liquefaction process, various co-liquefaction partners, such as the feedstock, the reaction intermediates, or the biocrude molecules, could get involved in the HTL of MSS from time to time. Their roles in the co-liquefaction reactions could be positive, negative, or have no effect.^40,42^ Herein, we selected three reaction temperatures (300, 320, and 340 °C), the appropriate temperatures for the highest biocrude yield from co-liquefaction of MSS with the three model biochemical compounds (soy oil, starch, and soy protein), to figure out whether there was a synergistic effect on the biocrude yield in co-liquefaction. The model feedstocks (soy oil, soy protein, and starch) were liquefied at their corresponding modified reaction temperature individually, with a holding time of 60 min and water content of 80%. The MSS also underwent the HTL process at the same three temperatures (300, 320, and 340 °C), with the same operating conditions. The biocrude yield and element content of the six HTL experiments are presented in Table 2.

**Table 2:** Biocrude yield and element content for the MSS and model feedstocks liquefied alone at 300, 320, and 340 °C

Temperature (°C)	Compounds	Biocrude yield (%)	Element content (%)					HHV (MJ kg^−1^)
			C	H	O	N	S	
300	Soy oil	94.87	82.31	8.55	9.14	0	0	37.75
	MSS	18.63	70.24	8.23	16.9	4.51	0.12	32.78
320	Starch	9.18	66.54	8.77	24.69	0	0	31.12
	MSS	19.21	68.43	7.94	17.72	5.67	0.24	31.82
340	Soy protein	32.55	73.12	7.44	12.36	6.87	0.21	33.39
	MSS	16.42	70.51	8.05	14.25	6.82	0.37	33.11

To investigate whether there is a synergistic effect, a comparison of the theoretical and actual values of biocrude yield was taken and presented in Table 3. As shown in Table 3, the co-liquefaction with soy oil showed the highest biocrude yield. Similar results were obtained for the co-liquefaction of swine manure and waste vegetable oil.^43^ However, due to the high conversion ratio of individual soy oil in the HTL process, the synergistic effect on the biocrude yield was not so impressive. Only a 6.73% improvement was observed according to the calculation. It seems that adding extra lipid compounds could increase the biocrude yield due to the high biocrude yield from the lipids themselves, rather than from intermolecular reactions between the MSS and soy oil. On the other hand, co-liquefaction of starch and the MSS presented a lower biocrude yield, however a marvelous synergistic effect on the biocrude yield was observed. The actual biocrude yield was 57.25% higher than that determined from theory. During the co-liquefaction process, there must have been plenty of reactions that occurred between the starch and the MSS, forming more compounds into co-liquefaction biocrude. However, because of the essential properties of the starch and the MSS, the significant synergistic effect could not promote the biocrude yield to a high value. But this synergistic effect could be used as evidence to investigate co-liquefaction of MSS and cellulose-rich biomass waste. The co-liquefaction of the MSS and soy protein also showed a milder synergistic effect, with an increase in the biocrude yield of 22.99%. According to the biochemical composition, the MSS feedstock is protein-rich and starch-rich. The synergistic effect could come from the Maillard reaction, which is the chemical reaction between amino acids and reducing sugars. The reactive carbonyl group of the sugar reacts with the nucleophilic amino group of the amino acid, and forms a complex mixture of poorly characterized heterocyclic molecules in the biocrude, as Yang^44^ described in the co-liquefaction of sugar and protein.

**Table 3:** Biocrude yield comparison and element content for the co-liquefaction reactions

Index	Oil + MSS	Starch + MSS	Protein + MSS
Reaction temperature (°C)	300.00	320.00	340.00

**Biocrude yield**			
Theoretical	56.75	14.20	24.49
Actual	60.57	22.33	30.12
Synergistic effect (%)	6.73	57.25	22.99

**Element content (%)**			
C	69.2	72.5	69.2
H	8.2	7.8	8.5
O^a^	17.8	14.9	13.7
N	4.5	4.2	8.4
S	0.3	0.6	0.2

**Element ratio**			
O/C	0.19	0.15	0.15
H/C	1.42	1.29	1.47
HHV (MJ kg^−1^)	32.34	33.29	33.34
Energy recovery (%)	82.21	48.23	55.25

**Element enrichment (%)**			
C	84.30	44.51	52.94
H	66.62	31.35	44.10
O	41.02	8.00	13.13
N	97.78	34.26	26.25
S	71.12	52.44	23.58

a Calculated by differences.

In conclusion, the co-liquefaction reactions of the MSS with a protein, saccharide, or lipid all showed a synergistic effect on the biocrude yield. However, where the synergistic effect came from was not clear. There could be intermolecular reactions.^45^ However, one cannot deny that the liquefaction process of the additional compounds might be catalytically promoted by the inorganic components in the MSS.^23^ Further analysis should be undertaken in the future, but this topic is beyond the scope of this study.

### 3.3 Thermogravimetric analysis (TGA) of the biocrude samples

The TGA experiments were carried out in a nitrogen atmosphere with a programmed heating operation, which could be regarded as a simulated miniature distillation. Hence, TGA has been widely used for estimating the boiling point distribution of the biocrude from HTL.^32,46^ In this section, we discuss the TGA experimental results, and the boiling point distributions of the obtained biocrude samples are listed in Table 4.

**Table 4:** The boiling point distribution of the co-liquefaction biocrude samples

Boiling point range (°C)	Biocrude fraction (%)			
	MSS	MSS + soy oil	MSS + soy protein	MSS + starch
50–150	15.29	33.21	8.10	13.39
150–200	23.44	18.94	13.47	12.37
200–250	18.38	10.85	13.43	9.51
250–300	9.86	4.63	18.49	12.36
300–350	2.12	2.88	13.21	6.88
350–500	15.06	10.54	28.10	12.24
>500	15.85	5.99	6.94	33.25

As shown in Table 4, regardless of the composition of the feedstock, the major boiling point was in the range of 150 to 500 °C. This result suggested that all the biocrude samples contained more than 65 wt% of volatiles, which showed the potential for further refining. However, adding organic compounds to the liquefaction of the MSS did affect the distribution in different ways. Adding lipids (soy oil) showed a most pleasing trend for more light oil-like fractions to appear in the co-liquefaction biocrude. The fraction of light oil compounds (with a boiling point of less than 350 °C) increased from 69.09 to 83.47%, showing a positive effect on light oil compound production. The result was not surprising because there are plenty of volatiles primordially existing in the soy oil feedstock, and they would convert into, or directly be regarded as, the HTL biocrude.^47^ However, the comparison between the biocrude samples before/after adding protein suggested that the promotional synergistic effect on the biocrude yield by adding soy protein did not appear to lead to the production of more light oil fractions. The light oil fraction decreased by 6% after co-liquefaction with soy protein. A possible explanation could be that the Maillard reactions that take place between hydrocarbons and proteins are able to form bigger molecules. Therefore, after adding the protein, small compounds could be converted into higher-vapor point compounds *via* polymerization reactions. On the other hand, although the co-liquefaction of starch and MSS showed a significant improving effect on the biocrude yield, it also showed a catastrophic behavior toward improving the boiling point distribution. Only 54.51% of the co-liquefaction biocrude molecules belonged to light oil. It seemed that intermolecular reactions in the starch to form alcohol and aldehyde structures could happen. Starches are firstly hydrolyzed to oligosaccharides, then hydrolyzed further to monosaccharides, and monosaccharides could be converted into furans.^48^ These sugar compounds could keep on reacting with other kinds of biomolecules. A large increase in fractions with a boiling point higher than 500 °C is notable after co-liquefaction with starch. The reason for this could be that these maltose, glucose, fructose and furfural derived compounds were hard to vaporise.^40^ Taking the biocrude yield, the synergistic effect, and the boiling distribution into consideration, the co-liquefaction of the MSS with the protein behaved well in all three indexes, especially when taking into account the price of lipid-rich and protein-rich biomass waste feedstock.^16,49,50^ Co-liquefaction of MSS with protein-rich biomass, such as microalgae, manure, and human feces, could be a good method to handle municipal waste disposal and renewable energy production.^1,24,51^

### 3.4 Molecular composition of the obtained biocrude

The GC-MS method was adopted for the analysis of the molecular composition of the biocrude samples. The main compounds identified by GC-MS are presented in Table 5. It should be noted that only chemical compounds with relative areas of more than 0.5% were selected for analysis. However, due to the final programmed temperature of the GC (250 °C), no more than half of the biocrude samples could be volatilized and detected by the GC, based on the TGA in Section 3.3. However, the qualitative comparison between different co-liquefaction biocrude samples can provide basic information about the molecular composition of the biocrude, especially the light fraction. To simplify the GC-MS analysis, we only checked and compared the presence of the identified compounds in the co-liquefaction biocrude. As shown in Table 5, the biocrude from co-liquefaction with soy oils showed a very similar collection of compounds identified by GC-MS to the biocrude from HTL of the MSS alone. After co-liquefaction, there was only one kind of new compound (saturated linear alkanes) in the soy oil co-liquefaction biocrude. The new compound that appeared, heptadecane, could have come from the hydrolysis of lipids. As Wang^32^ described previously, fatty acids produced from lipid hydrolyzation underwent decarboxylation reaction with amino acids in MSS then paraffin formed. Moreover, the appearance of fewer new compounds confirmed our previous conjecture in Section 3.2 that adding soy oil (lipid) had a less synergistic effect on the co-liquefaction of MSS. The co-liquefaction of protein or starch, on the other hand, provided several new kinds of components with more complex structures and functional groups (cyclohexanone, cyclopentanone, benzene, and indenone). These cyclic organic compounds with heterocyclic rings or benzene rings must come from intramolecular and intermolecular cyclization reactions.^33^ These cyclization reactions inevitably lead to the formation of compounds with more rings and heavier molecular weights. That could explain why there were less light oil fractions in the co-liquefaction biocrude with soy protein or starch. However, due to the limitations of current mass spectrum identification methods, we only provided some possible reaction pathways during the co-liquefaction process. More detailed characterization of the biocrude should be undertaken to further identify all the compounds to figure out the actual reactions during the co-liquefaction process for liquefaction mechanism analysis.

**Table 5:** Molecules identified from the co-liquefaction samples

Identified compounds	Bimolecular models			
	MSS	MSS + soy oil	MSS + soy protein	MSS + starch
Phenol	✓	✓	✓	✓
Phytol	✓	✓	✓	✓
Indole	✓	✓	✓	✓
Pyrrole	✓	✓	✓	✓
Piperidine	✓	✓	✓	✓
Hexadecanamide	✓	✓	✓	✓
Hexadecane		✓		
Heptadecane		✓		
Cyclohexanone			✓	✓
Cyclopentanone			✓	✓
Benzene				✓
Indenone				✓
Hexadecanoic acid	✓	✓	✓	✓

## 4 Conclusion

Co-liquefaction of MSS and three kinds of model feedstock could increase the biocrude yield by different degrees, and co-liquefaction with soy oil provided the highest biocrude yield of 60.57%. All the co-liquefaction processes showed a promotional synergistic effect on the biocrude yield and the starch feedstock promoted the most by 57.25%. TGA suggested co-liquefaction with protein or starch produced less light oil fractions and GC-MS indicated that this could be because of the formation of more cyclic organic compounds. Co-liquefaction of MSS with cellulose-rich or protein-rich biomass feedstock could be a promising method for biomass waste disposal and renewable energy production in the future.

## Author contributions

Wenjia Wang: methodology, investigation, data curation, writing – original draft, Hongbiao Du: conceptualization, validation, formal analysis, visualization, Yuanyuan Huang: conceptualization, investigation, Shaobo Wang: formal analysis, writing – original draft, Chang Liu: resources, writing – review & editing, Jie Li: resources, visualization, Jinglai Zhang: investigation, data curation, Shuai Lu: validation, supervision, project administration, Huansheng Wang: methodology, funding acquisition, Han Meng: writing – review & editing, supervision, project administration.

## Conflicts of interest

There are no conflicts of interest to declare.

## Supplementary Material
